# The Relation Between Quality of Life, Functional Impairment and Nutritional Status in Older People

**DOI:** 10.3390/healthcare14080978

**Published:** 2026-04-08

**Authors:** Gabriela Cristina Chelu, Cătălina Raluca Nuță, Ovidiu Lucian Băjenaru, Lidia Băjenaru, Gabriel Ioan Prada

**Affiliations:** 1 Faculty of Medicine, “Carol Davila” University of Medicine and Pharmacy, 050474 Bucharest, Romania; gabriela-cristina.chelu@umfcd.ro (G.C.C.); ovidiu.bajenaru@umfcd.ro (O.L.B.); gabriel.prada@umfcd.ro (G.I.P.); 2 National Institute of Gerontology and Geriatrics “Ana Aslan”, 011241 Bucharest, Romania; 3 Department of Communications, Applications, and Digital System, National Institute for Research and Development in Informatics—ICI Bucharest, 011455 Bucharest, Romania; lidia.bajenaru@ici.ro; 4 Department of Computer Science, Faculty of Automatic Control and Computers, National University of Science and Technology POLITEHNICA Bucharest, 060042 Bucharest, Romania; 5 Academy of Romanian Scientists, 050044 Bucharest, Romania

**Keywords:** quality of life, EQ-5D-5L, nutritional status, functional impairment

## Abstract

**Background/Objectives**: Autonomy and the ability to live independently are priority goals for older adults and are closely linked to healthy aging and quality of life (QoL). However, nutrition, although a crucial and modifiable determinant, remains undervalued. Cardiovascular diseases are highly prevalent in middle-aged and older adults and increase the risk of functional impairment, burdening the economy and limiting the patient’s autonomy. This study aimed to analyse the quality of life in older adults and its relationship with functional impairment and nutritional status. **Methods**: This was a cross-sectional study that included 359 patients with a mean age of 71.52 years who were admitted to the National Institute of Gerontology and Geriatrics “Ana Aslan”, between January 2024 and April 2025. Data were collected through interviews, medical records, and standardized instruments, including the Up and Go Test, Tinetti Test, Downton Fall Risk Index, and handgrip strength assessment. Quality of life was assessed using the EQ-5D-5L and the visual analog scale (VAS). **Results**: Nutritional status assessed using MNA showed significant moderate-to-strong correlations with EQ-5D-5L mobility (r = −0.326, *p* = 0.007 in the ≥80 years’ group), anxiety/depression (r = −0.544, *p* < 0.001 in the ≥80 years’ group), self-care (r = −0.271 to −0.311, *p* < 0.05 in patients over 65), and usual activities (r = −0.294, *p* = 0.016 in the ≥80 years’ group). In contrast, BMI showed moderate positive correlations with EQ-5D-5L pain/discomfort across all age groups (r = 0.365 to 0.524, *p* < 0.002). Functional assessment revealed strong negative correlations between EQ-5D-5L mobility and the Tinetti Test (r = −0.583 to −0.728, *p* < 0.001), with weaker correlations for pain/discomfort and anxiety/depression dimensions. While BMI-EQ-5D-5L pain/discomfort correlations were consistent across age groups, a stronger correlation was observed in the ≥80 years’ group for MNA-EQ-5D-5L anxiety/depression. **Conclusions**: In this exploratory cross-sectional study, MNA and BMI were associated with different quality of life domains. Lower MNA scores were more frequently associated with anxiety/depression and certain functional domains, particularly in the ≥80 years’ group, whereas higher BMI was more consistently associated with pain/discomfort across age groups.

## 1. Introduction

According to the World Health Organization (WHO), quality of life (QoL) is defined as the perception of an individual of their position in life in terms of the culture and value systems in which they live and according to their goals, expectations, standards, and concerns [1]. Lopez et al. showed that QoL in older adults may be improved by better health status, personal growth, and a stronger sense of purpose in life, particularly among those with mild depression or anxiety [2]. Many studies have shown that aging naturally diminishes health-related quality of life (HRQoL) through cell death and degeneration, affecting physical and mental functions while increasing disease susceptibility. Noto et al. highlighted the importance of preventive interventions in aging populations, particularly those aimed at improving lifestyle through dietary change and physical activity, with the long-term goal of reducing cardiovascular events and frailty [3].

Various instruments are used to evaluate QoL, the most common being EQ-5D-5L [4] and Short Form (36) Health Survey (SF-36) [5]. The EQ-5D-5L, launched in 2009, was developed by the EuroQoL Group and assesses five aspects of HRQoL: mobility, self-care, usual activities, pain/discomfort, and anxiety/depression. It also incorporates a visual analog scale (EQ-VAS) that offers a quantitative assessment of health outcomes derived from patients’ views. The SF-36 questionnaire consists of 36 questions that evaluate the physical, mental, and social health of an individual, from which HRQoL can be summarized into physical and mental component scores.

Cardiovascular diseases (CVDs) affect approximately 60% of individuals aged over 60 years and represent a major cause of mortality worldwide, with diagnosed cardiovascular disease associated with a three-times higher risk of developing frailty syndrome. Frailty syndrome is present in 25–62% of patients with cardiovascular disease and has been shown to predict mortality independently of age and disease severity [6]. Traditional disease-focused approaches often overlook patient-centered priorities such as physical function and maintenance of independence. A comprehensive understanding of how functional impairment and cardiovascular comorbidities combined influence quality of life in older adults remains limited, which highlights the need for integrated assessment approaches in the older population [7].

According to the World Health Organization (WHO), the priority should shift from merely increasing life expectancy to increasing the number of years lived without functional disability. This perspective emphasizes the urgency of reducing the socioeconomic burden associated with poor health in the final years of life and highlights the importance of preserving independence in older adults [8].

Arensberg et al. showed that autonomy and living at home are valuable to older adults and, in order to achieve these goals, healthy aging and a high QoL are needed. Although nutrition is an important and potentially modifiable determinant of healthy aging and QoL, it often remains underrecognized [9].

Nutritional status in the elderly population is a complex concept that includes dietary intake, anthropometric parameters, and functional reserve. Malnutrition is prevalent in the elderly population and has been associated with increased morbidity, functional decline and reduced QoL [10]. Among the available tools, body mass index (BMI) remains a widely used anthropometric indicator that allows stratification of nutritional risk across clinical settings but it does not account for changes in body composition, such as age-related loss of muscle mass, and it may also underestimate the nutritional risk for the patients with underlying causes such as chronic illness, depression, medication and social isolation [10].

To address the limitations of BMI, the Mini Nutritional Assessment (MNA) was specifically designed and validated for use in elderly patients across hospital, outpatient, and community settings. The MNA provides a rapid assessment of nutritional status and has been validated in multiple clinical settings worldwide, with a sensitivity of 96%, specificity of 98% and a predictive value of 97% [11]. Most importantly, the MNA detects malnutrition risk before serum albumin levels and body mass index begin to decrease, making it particularly useful for early identification of at-risk older adults [12]. The combined use of BMI and MNA provides a comprehensive nutritional assessment, balancing objective anthropometric data with broader clinical and functional dimensions.

The SF-36 is a versatile and widely validated measure of HRQoL, applicable to a wide range of chronic conditions—including cardiovascular [13], neurological [14], rheumatological [15], and nephrological diseases [16]—and designed to capture multidimensional aspects of physical and mental health [17].

In contrast to the broader domain structure of the SF-36, the EQ-5D-5L offers a more concise approach to the assessment of HRQoL. Through its five standardized dimensions—mobility, self-care, usual activities, pain/discomfort, and anxiety/depression—combined with the EQ visual analogue scale (EQ-VAS), it captures both functional health status and the individual’s overall perception of health, facilitating its use in clinical research [18] and health outcome evaluation [19].

Aidoud et al. showed that the high comorbidity burden among older adults with cardiovascular disease supports the need for adapted models of care for this vulnerable population. The high prevalence of geriatric conditions associated with CVD emphasizes the need for an integrated, multisystem approach [20].

The importance of a patient-centered approach was shown by Jeon et al. in their study, focusing on the importance of maintaining functionality, independence, QoL and dignity in older patients, especially in the decision-making process before applying routine recommendations from evidence-based guidelines [21].

In another study, Komalasari et al. showed that most patients with a history of CVD had a good quality of life based on environmental aspects, social interactions, physical and psychological status [22].

Therefore, the aim of this study is to evaluate the relationship between nutritional status and QoL among older adults with functional impairment to better understand factors negatively affecting QoL and to support physicians in their decision-making for their patients.

## 2. Methods

The study was conducted between January 2024 and April 2025 and included a total of 359 patients who were admitted to the National Institute of Gerontology and Geriatrics “Ana Aslan”, Bucharest, over the age of 40, with a mean age of 71.52 ± 8.944 years. The inclusion criteria were age ≥ 40 years and inpatient admission during the study period. The study excluded all patients under the age of 40 and all patients who did not provide consent to participate in the study. Each patient was informed of the study objectives, and the consent form was explained to eliminate any uncertainties about the research methodology; after which, the patient was requested to sign the consent form. All eligible patients admitted to the National Institute of Gerontology and Geriatrics during the study period were enrolled consecutively. Complete data were obtained for all the patients included in the final analysis; no missing data were recorded, and no imputation procedures were required. The protocol for this study was approved by the local ethics committee (Number 113/23.11.2023 & 231/04.04.2025).

The patients included in the study were divided into four groups: a control group, patients aged 40 to 64 years, and 3 study groups (patients between 65 and 74 years and 75 and 79 years, and over 80 years). The 40–64 years range was selected as a control group, in line with established age classification frameworks grounded in the United States Census and widely used in research, where middle-aged adults (approximately 40–64 years) are distinguished from the older adults (≥65 years) [23,24].

Demographic data (age, gender) and anthropometric data (weight, height) were collected from the observation sheet of each patient within the study timeframe. We also recorded the presence of heart failure (HF), ischemic heart disease (IHD), type 2 diabetes mellitus (T2DM), and osteoarthritis. Quality of life (QoL) was assessed using the EQ-5D-5L, which includes five domains (mobility, self-care, usual activities, pain/discomfort and anxiety/depression), each assessed across five levels (no problems, slight problems, moderate problems, severe problems and extreme problems) [4], and the EQ-VAS, a self-rated scale consisting of a horizontal line with values from 0 to 100, where 0 is the worst health state and 100 is the best health state [25]. Poor balance and reduced physical activity were assessed using the Tinetti Test (a score of 23 points or lower indicated impairment, and a score above 23 points was considered normal, so a score between 24 and 26 points was considered normal, between 19 and 23 represented a fall risk present, and lower than 19 points represented a high fall risk) [26]. Frailty-related mobility was assessed using the Up and Go Test, with values below 10 s considered normal and values of 10 s or more considered impaired [27,28]. Handgrip strength was also assessed [29], and fall risk was evaluated using the Downton Fall Risk Index (DFRI) (a score between 0 and 1 point is considered a lower risk, between 2 and 3 points a moderate risk, and equal and/or higher than 4 points is considered a high risk) [30]. Nutritional status was evaluated using body mass index (BMI) (underweight for BMI values below 18.5 kg/m^2^, normal weight for BMI values between 18.5 and 24.9 kg/m^2^, overweight for BMI values between 25.0 and 29.9 kg/m^2^, class I obesity for BMI values between 30.0 and 34.9 kg/m^2^, class II obesity (severe obesity) for BMI values between 35.0 and 39.9 kg/m^2^, and class III obesity (morbid obesity) for BMI values of 40.0 kg/m^2^ or higher) and the Mini Nutritional Assessment (MNA) (score between 24 and 30 points is considered a normal nutritional status and a score below 23.5 points is considered at risk for malnutrition) [31]. T2DM was diagnosed by the diabetologist prior to hospitalization or by the attending physician based on international criteria. HF and IHD were diagnosed by the cardiologist prior to hospitalization.

Osteoarthritis was diagnosed by the attending physician according to American College of Rheumatology criteria [32] or was established prior to hospitalization.

The analysis was performed using IBM SPSS Statistics 20. Continuous variables were expressed as mean ± standard deviation (SD), while categorical variables were expressed as frequencies and percentages. The chi-squared test was used to evaluate associations between categorical variables, and Spearman’s rho correlation coefficients were used to assess the relationship between ordinal and continuous variables. Statistical significance was set at *p* < 0.05. Because multiple correlation analyses were performed, statistical significance at *p* < 0.05 was interpreted as exploratory rather than confirmatory, particularly for isolated findings with borderline *p*-values.

## 3. Results

A total of 359 patients were enrolled in this study, with the general characteristics of the population summarized in Table 1. The mean age distribution showed that the majority of patients were in the age group of 65–74 years (40.4%), followed by an almost equal distribution between the age group of 75–79 years (20.9%) and ≤64 years (20.1%). The study population was predominantly female (82.2%). Although only 42.7% of the population was identified as obese, in contrast, a high proportion of the sample was found to have a normal nutritional status (80.6%), as determined through the MNA. Ischemic heart disease (IHD) was present in 30.9% of the study population, followed by heart failure (HF), identified in 21.4% of participants. Type 2 diabetes mellitus (T2DM) was diagnosed in 27.3% of the population, while prediabetes was identified in only 1.1% of patients. Osteoarthritis, an important factor for the risk of falls and lower quality of life, was documented in more than half of the sample (58.5%).

A progressive decline was observed in all measured parameters for functional assessment, as shown in Table 2. High fall risk (Downton Fall Risk Index) increased three times from the youngest age group to the oldest age group (27.8% vs. 77.6%, *p* < 0.0001). Tinetti Score revealed a decrease in balance and gait function, starting with normal performance from 81.9% to 32.8% (*p* < 0.001) in subjects from the age group ≤64 years versus ≥80 years. Mobility impairment (Up and Go Test) increased significantly with age (11.4% to 57.1%, *p* < 0.001), while handgrip strength diminished bilaterally: right arm measurements decreased from 24.4 ± 8.9 kg to 17.7 ± 7.6 kg (*p* < 0.001) and left arm from 22.4 ± 9.8 kg to 16.7 ± 8.6 kg (*p* < 0.001).

Overweight had the highest prevalence in the 75–79 age group (46.7%, *p* = 0.038), while normal weight had the highest prevalence in the ≥80 group (29.9%, *p* = 0.038) (Table 3). Severe obesity was absent in the age group ≥80 years. Although there are high overweight and obesity rates, MNA assessment revealed a decline in the nutritional status with age: normal nutrition status decreased from 87.5% to 62.7%, while malnutrition risk increased from 12.5% to 37.3% across age groups (*p* < 0.0007).

The frequencies and proportions of EQ-5D-5L by dimension and level in the overall study population are presented in Table 4. The highest frequency reported was “not affected” in usual activities (75.2%), followed by anxiety/depression (66.9%) and self-care (64.6%). At the “slightly affected” level, the highest frequency was reported for the pain/discomfort dimension (41.5%), followed by mobility (37.9%) and self-care (26.2%). The “extremely affected” level was slightly represented among the studied population; its frequency barely reaching 0.3% in the self-care and usual activities domain.

**Figure 1:**
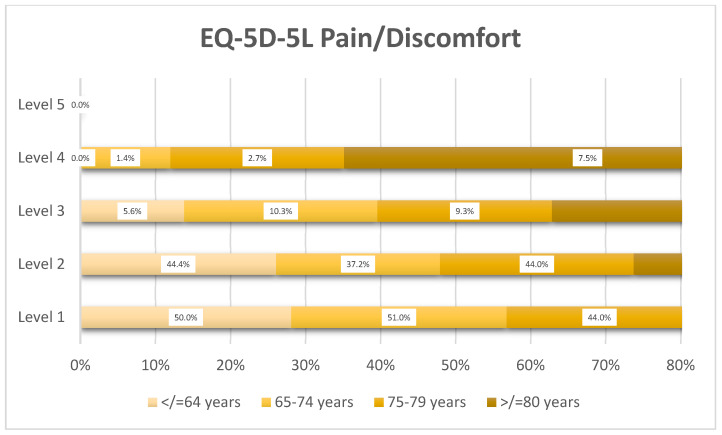
Distribution of EQ-5D-5L pain/discomfort levels by age group (*n* = 359).

Pain/discomfort severity increased progressively with age (Figure 1), with severe levels (Level 3–4) rising from 5.6% in the ≤64 years’ group to 22.4% in the ≥80 years’ group. No patients reported extreme pain/discomfort.

**Figure 2:**
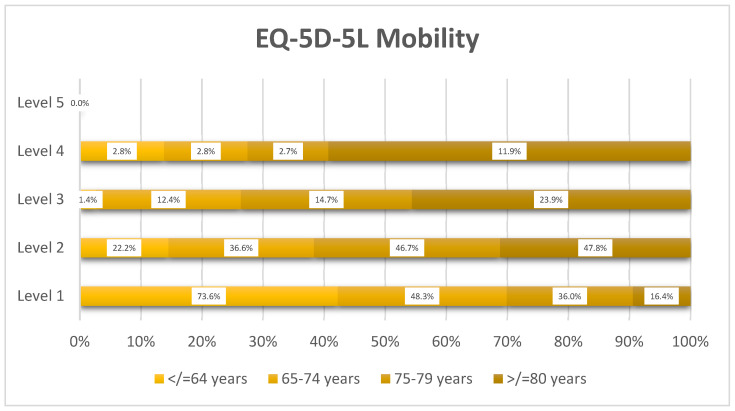
Distribution of EQ-5D-5L mobility levels by age group (*n* = 359).

Mobility impairment showed a pronounced age-related decline (Figure 2): the proportion with no problems had a decrease from 73.6% in the ≤64 years’ group to 16.4% in the ≥80 years’ group, while moderate-to-severe impairment (Level 3–4) increased from 4.2% to 35.8%.

**Figure 3:**
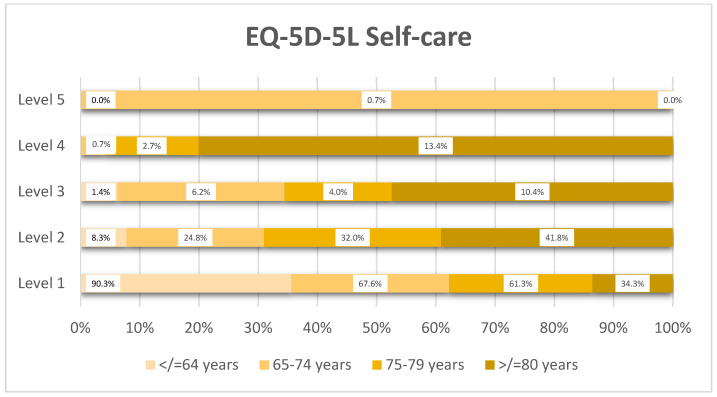
Distribution of EQ-5D-5L self-care levels by age group (*n* = 359).

Self-care dependency increased significantly with age (Figure 3): while 90.3% of patients in the ≤64 years’ group reported no problems, this percentage reduced to 34.3% in the ≥80 years’ group, with 13.4% of octogenarians reporting severe impairment.

**Figure 4:**
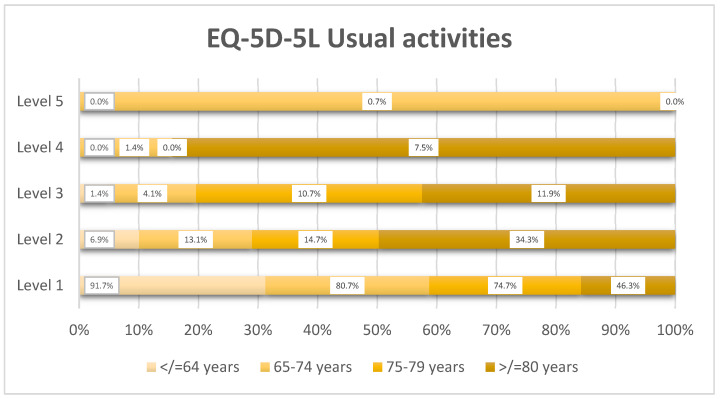
Distribution of EQ-5D-5L usual activities levels by age group (*n* = 359).

Limitations in usual activities followed a similar age-dependent pattern (Figure 4), with the proportion of patients reporting any impairment increasing from 8.3% in the ≤64 years’ group to 53.7% in the ≥80 years’ group. In the oldest group, moderate-to-severe problems accounted for 24.9%.

**Figure 5:**
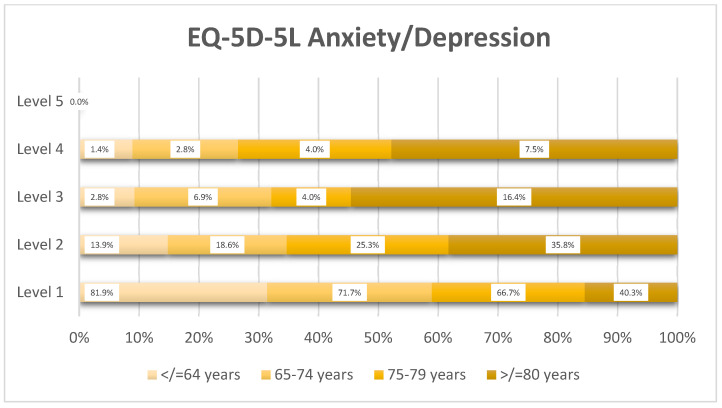
Distribution of EQ-5D-5L anxiety/depression levels by age group (*n* = 359).

Anxiety/depression burden was most prominent in the oldest age group (Figure 5): only 40.3% of patients ≥80 years reported no problems, compared with 81.9% in the ≤64 years’ group, with severe symptoms (Level 3–4) present in 23.9% of octogenarians.

**Figure 6:**
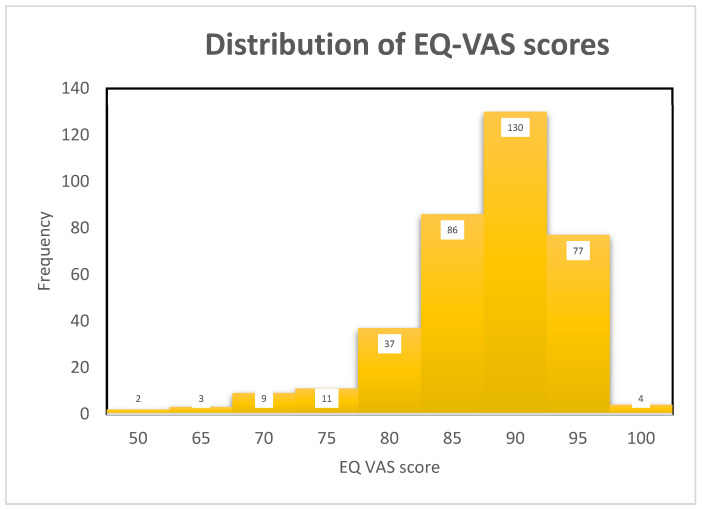
Frequency distribution of EQ-VAS scores (*n* = 359).

Despite the functional decline described above, self-perceived health status was high across the study population: 82.7% of participants scored ≥85 on EQ-VAS, and only 3.9% scored below 75 (Figure 6). This discrepancy between objective functional measures and subjective health perception is consistent with the multidimensional nature of self-rated health in older adults.

Exploratory age-stratified analyses suggested a negative association between MNA scores and the mobility domain of the EQ-5D-5L (Table 5). The clearest pattern was observed in the ≥80 years’ group (r = −0.326, *p* = 0.007), while a weaker association was found in the 65–74 years’ group (r = −0.166, *p* = 0.046). BMI showed only a weak positive association with mobility in the 65–74 years’ group (r = 0.196, *p* = 0.018).

BMI-based nutritional status demonstrated moderate positive correlations with the pain/discomfort domain of the EQ-5D-5L across all age groups (Table 6): ≤64 years (r = 0.523, *p* < 0.001), 65–74 years (r = 0.524, *p* < 0.001), 75–79 years (r = 0.497, *p* < 0.001) and ≥80 years (r = 0.365, *p* < 0.001). In contrast, MNA scores were not significantly correlated with pain/discomfort in any age group.

Negative associations were identified between MNA scores and the anxiety/depression domain of the EQ-5D-5L, with age-related variation (Table 7). The strongest association was observed in the ≥80 years’ group (r = −0.544, *p* < 0.001), while a moderate association was also found in the 65–74 years’ group (r = −0.301, *p* < 0.001). No significant correlations were found for BMI across age groups.

For patients aged over 65 years, MNA scores were negatively correlated with the self-care domain of the EQ-5D-5L (Table 8). The clearest association was observed in the 65–74 years’ group (r = −0.271, *p* = 0.001), while weaker associations were found in the 75–79 years’ group (r = −0.229, *p* = 0.048) and ≥80 years’ group (r = −0.311, *p* = 0.010). BMI was not significantly correlated with self-care in any age group.

Associations between MNA-defined nutritional status and the usual activities domain were observed only in selected age groups and should be interpreted cautiously (Table 9). A weak association was found in the 65–74 years’ group (r = −0.174, *p* = 0.036), and a weak-to-moderate association in the ≥80 years’ group (r = −0.294, *p* = 0.016). No significant correlations were found for BMI across age groups.

Among functional performance measures, the Tinetti Test showed the strongest and most consistent association with EQ-5D-5L mobility domain across all age groups (r = −0.583 to −0.728, *p* < 0.001; Table 10), underlining the central role of balance and gait in determining perceived mobility limitations. The Up and Go Test corroborated this pattern with moderate positive correlations in most groups. Handgrip strength also showed relevant associations, particularly in the 75–79 years’ group (right arm: r = −0.526, *p* < 0.001), suggesting that upper limb weakness may contribute to reduced mobility-related QoL in this age group.

Functional performance parameters showed only modest associations with EQ-5D-5L pain/discomfort domain (Table 11). The Tinetti Test reached weak to moderate significance across age groups (r = −0.298 to −0.377, *p* < 0.011), whereas the Downton Fall Risk Index was non-significant. These attenuated correlations, compared with those observed for mobility and self-care, suggest that pain/discomfort in this population study is driven more by anthropometric factors (notably BMI) than by functional performance on its own.

Associations between functional performance and EQ-5D-5L anxiety/depression domain were age-dependent and inconsistent across groups (Table 12). No significant correlations emerged in the youngest group. The most consistent pattern of associations emerged in the ≥80 years’ group, where the Tinetti Test (r = −0.350, *p* = 0.004), Up and Go Test (r = 0.324, *p* = 0.010), and handgrip strength (right arm: r = −0.394, *p* = 0.001) all showed moderate associations, suggesting that physical frailty and psychological distress converge particularly in the oldest age group.

The Tinetti Test showed the strongest and most consistent correlations with EQ-5D-5L self-care domain across all age groups (r = −0.530 to −0.681, *p* < 0.001; Table 13), with the ≥80 years’ group showing the strongest association (r = −0.681). This finding highlights poor balance and gait as important contributors to dependency in activities of daily living across age groups. The Up and Go Test supported this pattern, with moderate correlations in the 65–74 and ≥80 years’ groups.

Associations between functional performance and the usual activities domain of EQ-5D-5L followed a consistently similar pattern to mobility and self-care domains (Table 14). The Tinetti Test reached significance across three age groups, with the strongest association in the ≥80 years’ group (r = −0.481, *p* < 0.001), while the Downton Fall Risk Index was non-significant across all groups. These findings indicate that gait and balance are closely associated with QoL limitations across multiple domains and may be particularly relevant from a clinical perspective.

## 4. Discussion

The main objectives of the present study were to analyse the complex relationships between nutritional status, functional performance, and QoL in a group of elderly patients who were evaluated in a geriatrics center.

While BMI showed consistent positive correlations with EQ-5D-5L pain/discomfort domain across all age groups (r = 0.365–0.524), most likely reflecting the mechanical load on joints and musculoskeletal structures, in contrast, MNA showed no significant association with EQ-5D-5L pain/discomfort domain. In a study published by Rat et al. [33], lower levels of QoL evaluated using the SF-36 questionnaire were linked to higher fat mass index, fat mass percentage, and lower Trunk Fat Mass to Limb Fat Mass; another recent study published by Song et al. [34] where they evaluated QoL using the HRQoL instrument with eight items (HINT-8), developed by Jo MW in 2014 [35] and validated in 2016 [36], found that the HINT-8 climbing stairs domain was positively associated with obesity, due to mechanical loading on joints and cardiorespiratory burden, and therefore, reduced QoL in the older population is closely linked to a decline in physical function. By looking into other studies that do not evaluate QoL, but various characteristics of pain, we can observe a direct causal connection between BMI and knee pain, hip pain and back pain identified by Chen et al. in their study [37]. In another study, Johnson et al. [38] reported positive associations between higher body fat mass, BMI, and widespread pain, supporting our findings. Zhong et al. [39] have shown in their study that chronic pain experienced by obese individuals may be due to the mechanical load of carrying the extra weight. In a systematic review and meta-analysis, it has been shown that patients with excess weight or obesity are more likely to report greater intensities of pain than normal weight individuals, supporting the idea of weight loss as a key intervention for pain management [40].

Lower MNA scores were associated with worse anxiety/depression and some functional EQ-5D-5L domains in specific age groups, more notably in the ≥80 years’ group (r = −0.544, *p* < 0.001), suggesting that a normal nutritional status becomes increasingly important for psychological well-being in older age, but these associations should be interpreted cautiously, given the cross-sectional design and the potential contribution of comorbidity burden. Stratidaki et al. reported a statistically significant correlation between MNA and geriatric depression, mental state, functional independence, and QoL evaluated with the WHOQOL-BREF scale; therefore, maintaining good mental health in the older community might be a good strategy to reduce the risk of malnutrition [41].

Higher BMI showed the most consistent unadjusted association with the pain/discomfort domain across age groups; however, this finding may also reflect the burden of osteoarthritis and other comorbidities in this inpatient population.

In our study, the strong correlations between performance parameters and EQ-5D-5L dimensions, especially for mobility and self-care, highlight the important role of physical function in maintaining a good QoL. The Tinetti Test has proven the strongest negative association with EQ-5D-5L mobility domain (r = −0.583 to −0.728), showing that balance and gait are fundamental determinants of perceived mobility limitations, as also reported in a 2025 study by Hayati et al. [42].

Oh et al. [43] reported significant associations between exercise type and the EQ-5D-5L self-care and mobility domains, with better QoL observed in older adults who exercised than in those who did not. These findings support the close relationship between functional independence and nutritional status and emphasize the value of including both nutritional screening and functional assessment in the comprehensive geriatric evaluation. Addressing impairments in these domains may help improve health outcomes and QoL in older adults, as also suggested by Reza et al. [44] and Murillo-Llorente et al. [45].

Significant correlations were observed between MNA and EQ-5D-5L self-care domain in patients over 65 years, suggesting that nutritional deficits become increasingly relevant to functional independence only after this age. This age-dependent pattern suggests that nutritional status is closely linked to physiological changes associated with aging, including sarcopenia, reduced physiological reserve, and multiple comorbidities. In our study, the risk of malnutrition was observed in the ≥80 years’ group (37.3%), which aligns with Miletic et al.’s findings about the vulnerability and necessity of improving health outcomes in this age group [46].

In our study, 82.7% of the participants reported high self-perceived health status (EQ-VAS ≥ 85), suggesting a high self-perceived health status despite an age-related functional decline in mobility, balance, and handgrip strength. This positive perception of health status is consistent with the findings in Machόn et al.’s study [47] of the multidimensional nature of self-perceived health in the older population.

The associations observed in our study between nutritional status and certain EQ-5D-5L domains may indicate that nutritional vulnerability coexists with reduced functional independence and poorer psychological well-being in older adults. However, because the present analyses were cross-sectional and unadjusted, these findings should not be interpreted as evidence that nutritional interventions would necessarily improve these outcomes, as suggested in Dowling et al.’s review [48]. Rather, they support the value of considering nutritional assessment as part of a broader, multidimensional geriatric evaluation.

Similarly, the observed associations between functional performance measures and the anxiety/depression domain, particularly in participants over 75 years, should be interpreted cautiously. These findings may reflect a complex coexistence of reduced mobility, frailty, psychological burden, and comorbidity in older adults, rather than a direct or independent relationship.

In a systematic review, Muñoz Pinto et al. [49] showed that interventions based on physical training could possibly increase the levels of self-esteem and decrease depressive symptoms in older adults. Also, Zhang et al. [50] concluded that physical activity plays a very important role in the active aging approach, as it substantially contributes to improving physical performance, supporting psychological wellness, and improving, in general, QoL in older people.

### Study Limitations

The design of the study (cross-sectional) prevents establishing a cause-and-effect relationship, meaning we cannot establish if poor nutritional status leads to a decline in QoL or the other way around. The population in this study displayed a significant female representation, reflecting the tendency of women to seek medical care more frequently. This aspect limits generalizability to the male population.

Also, the patients enrolled in this study were all consecutive patients admitted during the study period, regardless of their diagnosis, and therefore the resulting subgroups were heterogeneous and variable in size, limiting the applicability of multivariate modeling. Future longitudinal studies should employ adjusted regression analyses to further explore the associations identified in this study.

The study population consisted of patients from a single geriatric inpatient center. Hospitalized older adults typically present with a higher burden of morbidity and functional dependence than community-dwelling individuals, limiting the generalizability of our findings.

Considering these aspects, longitudinal studies are needed to further identify potential bidirectional effects between nutritional parameters and QoL outcomes.

Because no adjusted multivariable analyses were performed, the present study cannot determine whether the reported associations are independent of age, sex, cardiovascular comorbidity burden, or osteoarthritis.

In addition, because multiple correlation analyses were performed, some age-specific associations may reflect chance findings and should therefore be interpreted cautiously.

Furthermore, this study was not pre-registered, which we acknowledge as a limitation.

## 5. Conclusions

This study suggests that nutritional status, functional performance, and quality of life are interrelated in older adults through several exploratory associations. Higher BMI was more consistently associated with the EQ-5D-5L pain/discomfort domain across age groups, whereas lower MNA scores were more often associated with anxiety/depression and certain functional domains, particularly in the ≥80 years’ group. Functional performance, especially gait and balance measures, was also associated with mobility and self-care domains.

Because the study was cross-sectional and the analyses were unadjusted, these findings should be interpreted cautiously and should not be considered evidence of independent effects, prediction, superiority, or clinical effectiveness. The observed associations may partly reflect differences in comorbidity burden, including osteoarthritis, ischemic heart disease, heart failure, and type 2 diabetes mellitus. Further longitudinal studies with adjusted multivariable analyses are needed to clarify the independent relationships between nutritional status, functional impairment, and quality of life in older adults.

## Figures and Tables

**Table 1:** General characteristics of the population (*n* = 359).

Characteristic	Category	Total (*n* = 359) *n* (%)	Mean ± SD
Age	≤64 years	72 (20.1)	71.52 ± 8.944
	65–74 years	145 (40.4)	
	75–79 years	75 (20.9)	
	≥80 years	67 (18.7)	
Sex	Female	295 (82.2)	-
BMI	Obesity	153 (42.7)	29.86 ± 5.472
MNA	Normal	289 (80.6)	25.47 ± 3.010
HF	Yes	77 (21.4)	-
IHD	Yes	111 (30.9)	-
Arthrosis	Yes	210 (58.5)	-
T2DM	Yes	98 (27.3)	-
	Prediabetes	4 (1.1)	-

**Table 2:** Distribution of balance, mobility, and handgrip strength assessment by age group (*n* = 359).

Age		≤64 Years	*p*-Value	65–74 Years	*p*-Value	75–79 Years	*p*-Value	≥80 Years	*p*-Value
		*n* (%)		*n* (%)		*n* (%)		*n* (%)	
Downton Fall Risk Index	Lower risk	9 (12.5%)	<0.0001	12 (8.3%)	<0.0001	1 (1.3%)	<0.0001	4 (6%)	<0.0001
	Moderate risk	43 (59.7%)	<0.0001	74 (51%)	<0.0001	30 (40%)	<0.0001	11 (16.4%)	<0.0001
	High risk	20 (27.8%)	<0.0001	59 (40.7%)	<0.0001	44 (58.7%)	<0.0001	52 (77.6%)	<0.0001
Tinetti Score	Normal balance and gait	59 (81.9%)	<0.001	91 (62.8%)	<0.001	37 (49.3%)	<0.001	22 (32.8%)	<0.001
	Fall risk present	10 (13.9%)	<0.001	35 (24.1%)	<0.001	31 (41.3%)	<0.001	25 (37.3%)	<0.001
	High fall risk	3 (4.2%)	<0.001	19 (13.1%)	<0.001	7 (9.3%)	<0.001	20 (29.9%)	<0.001
Up and Go Test	Impaired mobility	8 (11.4%)	<0.001	40 (28.0%)	<0.001	32 (43.8%)	<0.001	36 (57.1%)	<0.001
	Normal mobility	62 (88.6%)	<0.001	103 (72.0%)	<0.001	41 (56.2%)	<0.001	27 (42.9%)	<0.001
Handgrip Strength	Right arm (mean ± SD)	24.4 (±8.9)	<0.001	23.2 (±8.9)	<0.001	19.7 (±7.8)	<0.001	17.7 (±7.6)	<0.001
	Left arm (mean ± SD)	22.4 (±9.8)	<0.001	20.9 (±8.2)	<0.001	18.5 (8.4)	<0.001	16.7 (±8.6)	<0.001

**Table 3:** Distribution of BMI categories and nutritional status by age group (*n* = 359).

Age		≤64 Years	*p*-Value	65–74 Years	*p*-Value	75–79 Years	*p*-Value	≥80 Years	*p*-Value
		*n* (%)		*n* (%)		*n* (%)		*n* (%)	
BMI	Underweight	0 (0%)	0.038	1 (0.7%)	0.038	1 (1.3%)	0.038	1 (1.5%)	0.038
	Normal weight	11 (15.3%)	0.038	17 (11.7%)	0.038	11 (14.7%)	0.038	20 (29.9%)	0.038
	Overweight	25 (34.7%)	0.038	61 (42.1%)	0.038	35 (46.7%)	0.038	23 (34.3%)	0.038
	Class I obesity	22 (30.6%)	0.038	32 (22.1%)	0.038	15 (20%)	0.038	20 (29.9%)	0.038
	Class II obesity	11 (15.3%)	0.038	24 (16.6%)	0.038	10 (13.3%)	0.038	3 (4.5%)	0.038
	Class III obesity	3 (4.2%)	0.038	10 (6.9%)	0.038	3 (4%)	0.038	0 (0%)	0.038
MNA	Normal nutritional status	63 (87.5%)	0.0007	122 (84.1%)	0.0007	62 (82.7%)	0.0007	42 (62.7%)	0.0007
	Risk for malnutrition	9 (12.5%)	0.0007	23 (15.9%)	0.0007	13 (17.3%)	0.0007	25 (37.3%)	0.0007

**Table 4:** EQ-5D-5L frequencies and proportions reported by dimension and level.

EQ-5D-5L	Mobility	Self-Care	Usual Activities	Pain/Discomfort	Anxiety/Depression
	*n* (%)	*n* (%)	*n* (%)	*n* (%)	*n* (%)
Level 1 (not affected)	181 (44.8)	232 (64.6)	270 (75.2)	165 (46)	240 (66.9)
Level 2 (slightly affected)	136 (37.9)	94 (26.2)	58 (16.2)	149 (41.5)	80 (22.3)
Level 3 (moderately affected)	46 (12.8)	20 (5.6)	23 (6.4)	36 (10)	26 (7.2)
Level 4 (severely affected)	16 (4.5)	12 (3.3)	7 (1.9)	9 (2.5)	13 (3.6)
Level 5 (extremely affected)	0 (0)	1 (0.3)	1 (0.3)	0 (0)	0 (0)

**Table 5:** Nutritional status and EQ-5D-mobility.

EQ-5D-Mobility		Age			
		≤64 Years	65–74 Years	75–79 Years	≥80 Years
MNA	correlation	−0.138	−0.166	−0.185	−0.326
	*p*-value	0.247	0.046	0.113	0.007
BMI	correlation	0.150	0.196	0.087	−0.115
	*p*-value	0.208	0.018	0.456	0.352

Note: Spearman’s rank correlation coefficient.

**Table 6:** Nutritional status and EQ-5D-pain/discomfort.

EQ-5D-Pain/Discomfort		Age			
		≤64 Years	65–74 Years	75–79 Years	≥80 Years
MNA	correlation	−0.018	0.047	0.172	−0.161
	*p*-value	0.879	0.575	0.139	0.194
BMI	correlation	0.523	0.524	0.497	0.365
	*p*-value	<0.001	<0.001	<0.001	0.002

Note: Spearman’s rank correlation coefficient.

**Table 7:** Nutritional status and EQ-5D-anxiety/depression.

EQ-5D-Anxiety/Depression		Age			
		≤64 Years	65–74 Years	75–79 Years	≥80 Years
MNA	correlation	−0.136	−0.301	−0.164	−0.544
	*p*-value	0.255	<0.001	0.160	<0.001
BMI	correlation	−0.073	0.026	−0.049	−0.140
	*p*-value	0.541	0.755	0.676	0.258

Note: Spearman’s rank correlation coefficient.

**Table 8:** Nutritional status and EQ-5D-self-care.

EQ-5D-Self-Care		Age			
		≤64 Years	65–74 Years	75–79 Years	≥80 Years
MNA	correlation	−0.016	−0.271	−0.229	−0.311
	*p*-value	0.896	0.001	0.048	0.010
BMI	correlation	−0.082	0.112	0.108	−0.023
	*p*-value	0.494	0.179	0.357	0.851

Note: Spearman’s rank correlation coefficient.

**Table 9:** Nutritional status and EQ-5D-usual activities.

EQ-5D-Usual Activities		Age			
		≤64 Years	65–74 Years	75–79 Years	≥80 Years
MNA	correlation	0.114	−0.174	−0.083	−0.294
	*p*-value	0.341	0.036	0.477	0.016
BMI	correlation	0.128	0.091	0.001	−0.069
	*p*-value	0.285	0.279	0.992	0.577

Note: Spearman’s rank correlation coefficient.

**Table 10:** Functional performance parameters and EQ-5D-mobility.

EQ-5D-Mobility										
Age	Up and Go Test		Tinetti Test		Downton Fall Risk Index		Handgrip Strength			
							Right Arm		Left Arm	
	Correlation	*p*-Value	Correlation	*p*-Value	Correlation	*p*-Value	Correlation	*p*-Value	Correlation	*p*-Value
≤64 years	0.415	<0.001	−0.728	<0.001	0.328	0.005	−0.332	0.004	−0.356	0.002
65–74 years	0.416	<0.001	−0.648	<0.001	0.198	0.017	−0.137	0.102	−0.099	0.235
75–79 years	0.221	0.061	−0.583	<0.001	0.180	0.122	−0.526	<0.001	−0.469	<0.001
≥80 years	0.454	<0.001	−0.694	<0.001	0.182	0.140	−0.427	<0.001	−0.247	0.044

Note: Spearman’s rank correlation coefficient.

**Table 11:** Functional performance parameters and EQ-5D-pain/discomfort.

EQ-5D-Pain/Discomfort										
Age	Up and Go Test		Tinetti Test		Downton Fall Risk Index		Handgrip Strength			
							Right Arm		Left Arm	
	Correlation	*p*-Value	Correlation	*p*-Value	Correlation	*p*-Value	Correlation	*p*-Value	Correlation	*p*-Value
≤64 years	0.113	0.351	−0.299	0.011	0.047	0.698	−0.251	0.034	−0.253	0.032
65–74 years	0.296	<0.001	−0.298	<0.001	0.095	0.255	−0.060	0.473	−0.044	0.598
75–79 years	0.225	0.056	−0.377	0.001	0.081	0.488	−0.290	0.011	−0.328	0.004
≥80 years	0.316	0.012	−0.334	0.006	0.162	0.189	−0.073	0.559	−0.024	0.846

Note: Spearman’s rank correlation coefficient.

**Table 12:** Functional performance parameters and EQ-5D-anxiety/depression.

EQ-5D-Anxiety/Depression										
Age	Up and Go Test		Tinetti Test		Downton Fall Risk Index		Handgrip Strength			
							Right Arm		Left Arm	
	Correlation	*p*-Value	Correlation	*p*-Value	Correlation	*p*-Value	Correlation	*p*-Value	Correlation	*p*-Value
≤64 years	−0.061	0.617	−0.053	0.659	0.170	0.152	−0.055	0.644	−0.073	0.543
65–74 years	0.233	0.005	−0.214	0.010	0.041	0.629	−0.168	0.044	−0.222	0.007
75–79 years	0.011	0.925	−0.120	0.306	0.110	0.347	−0.304	0.008	−0.372	0.001
≥80 years	0.324	0.010	−0.350	0.004	0.196	0.111	−0.394	0.001	−0.262	0.032

Note: Spearman’s rank correlation coefficient.

**Table 13:** Functional performance parameters and EQ-5D-self-care.

EQ-5D-Self-Care										
Age	Up and Go Test		Tinetti Test		Downton Fall Risk Index		Handgrip Strength			
							Right Arm		Left Arm	
	Correlation	*p*-Value	Correlation	*p*-Value	Correlation	*p*-Value	Correlation	*p*-Value	Correlation	*p*-Value
≤64 years	0.244	0.042	−0.611	<0.001	0.301	0.010	−0.112	0.350	−0.272	0.021
65–74 years	0.402	<0.001	−0.598	<0.001	0.177	0.034	−0.094	0.260	−0.098	0.239
75–79 years	0.193	0.101	−0.530	<0.001	0.161	0.168	−0.323	0.005	−0.374	0.001
≥80 years	0.369	0.003	−0.681	<0.001	0.263	0.032	−0.229	0.063	−0.141	0.253

Note: Spearman’s rank correlation coefficient.

**Table 14:** Functional performance parameters and EQ-5D-usual activities.

EQ-5D-Usual Activities										
Age	Up and Go Test		Tinetti Test		Downton Fall Risk Index		Handgrip Strength			
							Right Arm		Left Arm	
	Correlation	*p*-Value	Correlation	*p*-Value	Correlation	*p*-Value	Correlation	*p*-Value	Correlation	*p*-Value
≤64 years	0.072	0.553	−0.285	0.015	0.096	0.425	−0.054	0.650	−0.338	0.004
65–74 years	0.200	0.016	−0.280	0.001	0.042	0.616	−0.031	0.712	−0.087	0.299
75–79 years	0.037	0.759	−0.228	0.049	0.042	0.721	−0.318	0.005	−0.345	0.002
≥80 years	0.299	0.017	−0.481	<0.001	0.072	0.560	−0.220	0.074	−0.154	0.212

Note: Spearman’s rank correlation coefficient.

## Data Availability

The data presented in this study are available on request from the corresponding author. The datasets presented in this article are not readily available because the authors do not own the database used in the presented statistics. They requested access to the database from the owner, the institution that conducted the study. The owner decided to keep the database private.
